# Treatment of surgical brain injury by immune tolerance induced by intrathymic and hepatic portal vein injection of brain antigens

**DOI:** 10.1038/srep32030

**Published:** 2016-08-24

**Authors:** Weijian Yang, Yong Liu, Baolong Liu, Huajun Tan, Hao Lu, Hong Wang, Hua Yan

**Affiliations:** 1 Graduate School of Tianjin Medical University, Tianjin 300070, China; 2 Department of Neurosurgery, Tianjin Huanhu Hospital, Tianjin 300060, China; 3 Department of Ultrasonography, Tianjin Huanhu Hospital, Tianjin 300060, China; 4 Tianjin Key Laboratory of Cerebral Vascular and Neurodegenerative Diseases, Tianjin 300060, China

## Abstract

Surgical brain injury (SBI) defines complications induced by intracranial surgery, such as cerebral edema and other secondary injuries. In our study, intrathymic and hepatic portal vein injection of allogeneic myelin basic protein (MBP) or autogeneic brain cell suspensions were administered to a standard SBI model. Serum pro-inflammatory IL-2, anti-inflammatory IL-4 concentrations and the CD4^**+**^T/CD8^**+**^T ratio were measured at 1, 3, 7, 14 and 21 d after surgery to verify the establishment of immune tolerance. Furthermore, we confirmed neuroprotective effects by evaluating neurological scores at 1, 3, 7, 14 and 21 d after SBI. Anti-Fas ligand (FasL) immunohistochemistry and TUNEL assays of brain sections were tested at 21 d after surgery. Intrathymic injections of MBP or autogeneic brain cell suspensions functioned by both suppressing secondary inflammatory reactions and improving prognoses, whereas hepatic portal vein injections of autogeneic brain cell suspensions exerted a better effect than MBP. Intrathymic and hepatic portal vein injections of MBP had equal effects on reducing secondary inflammation and improving prognoses. Otherwise, hepatic portal vein injections of autogeneic brain cell suspensions had better outcomes than intrathymic injections of autogeneic brain cell suspensions. Moreover, the benefit of injecting antigens into the thymus was outweighed by hepatic portal vein injections.

The central nervous system (CNS), especially the encephalon, is isolated from the immune system by the blood-brain barrier^1^. Disruption of the blood-brain barrier caused by a neurosurgical operation could result in inflammatory reactions in the CNS, which is known as surgical brain injury (SBI)^2^. The main function of the blood-brain barrier is to provide the optimal microenvironment for proper neuronal function^3^. After SBI or traumatic brain injury (TBI) destroys the blood-brain barrier, antigens of the brain tissue are released into systemic circulation. Therefore, the severity of blood-brain barrier destruction can be accurately indicated by anti-S-100 antibody concentrations^4^. Immune cells, cytokines, chemokines, and other inflammatory mediators are attracted to injury sites, which induces neural excitotoxicity, oxidative stress, mitochondrial dysfunction, and increased secondary inflammation^5^.

Current treatments for secondary inflammation include non-specific diuretics, anti-inflammatory agents, mild hypothermia therapy and immunosuppressive agents. Although these treatments have advantages for neural protection, they are likely to cause infection and tumors^6^. Fu Y *et al*. reviewed that immune interventions can reduce edema, apoptosis and brain atrophy in animal models of intracerebral hemorrhage^7^. By instilling antigen to the nasopharynx to induce immune tolerance, Ayer RE *et al*. achieved marked effects in the treatment of brain injury^8^. Huang H *et al*. reported that a combination of intrathymic and intravenous injections of mesenchymal stem cells can prolong the survival of rat cardiac allografts^9^. Studies of liver transplantations have found that some hosts were tolerant to grafts and no longer needed immunosuppressive agents after liver transplantation^10^,^11^. Therefore, we compared the efficiency of administration of MBP and autologous brain cell suspensions via the thymus and hepatic portal vein in inducing tolerance.

The thymus is crucially important for building central immune tolerance^12^. T lymphocytes in the thymus obtain MHC restriction via positive selection and obtain auto-tolerance via negative selection^13^. Autoimmune regulators together with other transcription factors regulate the transcription of endogenous genes in the thymus. The products are presented to T lymphocytes in the thymus to induce immune tolerance^14^. Dendritic cells, Kupffer cells, liver sinusoidal endothelial cells and hepatic stellate cells are among the specialized antigen-presenting cells that present antigens to T cells to participate in T cell apoptosis, anergy or the differentiation into regulatory T cells^15^. Li F *et al*. reviewed that the liver can served as a “school”. In that school, liver APCs serve as the “teachers” who “educate” circulating immune cell “students” to induce immune tolerance^16^. Since 1969, when Calne and his colleagues first discovered that a mismatched major histocompatibility complex (liver) could be tolerated by the host without immunodepressants^17^, inducing graft tolerance has become a pinnacle of pursuit. Therefore, the liver immune system plays an important role in the formation of immune tolerance.

MBP is the second most abundant protein after proteolipid protein in CNS^18^. Furthermore, MBP has been commercialized and is convenient use in for clinical treatments. Autogeneic brain cell suspensions contain various mixed antigens. In clinical treatments, a craniotomy must be performed to rescue patients who have suffered traumatic brain injury, especially patients with severe brain injury. To protect patients from infections, ruptured and necrotic brain tissue must be removed. Thus, this ruptured brain tissue can be exploited to prepare autogeneic brain cell suspensions for injection. This “waste utilization” can be used to develop new technologies and clinical treatments. Consequently, we sought to induce tolerance via intrathymic and hepatic portal vein injections of MBP or autogeneic brain cell suspensions containing equal dose antigens to determine whether they could decrease secondary inflammatory reactions.

## Results

### Serum Cytokine concentrations (pg/ml)

#### One-way ANOVA

##### Serum pro-inflammatory cytokine IL-2 concentration

**Figure 1:**
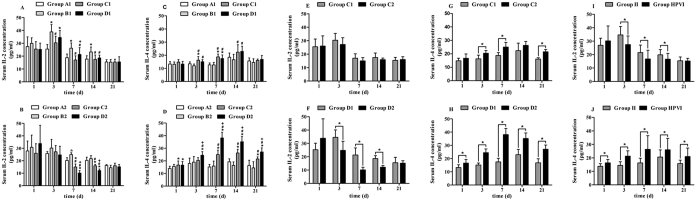
Serum cytokine concentration. In (**A–H**), Group A1: sham, Group A2: sham; Group B1: SBI+ intrathymic injection of normal saline; Group B2: SBI+ hepatic portal vein injection of normal saline; Group C1: SBI+ intrathymic injection of MBP; Group C2: SBI+ hepatic portal vein injection of MBP; Group D1: SBI+ intrathymic injection of autogeneic brain cell suspension; Group D2: SBI+ hepatic portal vein injection of autogeneic brain cell suspension. In (**I,J**), Group II: intrathymic injection group comprising Group B1 + Group C1 + Group D1; Group HPVI: hepatic portal vein injection group comprising Group B2 + Group C2 + Group D2. IL-2 and IL-4 concentrations were measured postoperatively at 1, 3, 7, 14 and 21 d. (**A,B**) Show pro-inflammatory cytokine IL-2 concentrations in Group A1/A2, Group B1/B2, Group C1/C2, and Group D1/D2. (**C,D**) Show anti-inflammatory cytokine IL-4 concentrations in Group A1/A2, Group B1/B2, Group C1/C2, and Group D1/D2. *P < 0.05 vs Group A1 (A2); ^#^*P* < 0.05 vs Group B1 (B2); ^+^*P* < 0.05 vs Group C1 (C2). (**E,F**) Show pro-inflammatory cytokine IL-2 concentrations in Groups C1 and C2 and in Groups D1 and D2, respectively. (**G,H**) Show anti-inflammatory cytokine IL-4 concentrations in Groups C1 and C2 and in Groups D1 and D2, respectively. *P < 0.05. (**I**) Shows pro-inflammatory cytokine IL-2 concentrations; (**J**) Shows anti-inflammatory cytokine IL-4 concentrations. *P < 0.05.

Compared with Group B1, IL-2 concentration was markedly decreased postoperatively in Group C1 at 3, 7 and 14 d and also decreased postoperatively in Group D1 at 7 and 14 d (*P* < 0.05). Furthermore, the concentrations of IL-2 in Group C1 and Group D1 were significantly different on the 7^th^ day (*P* < 0.05) (Fig. 1A). This result demonstrated that intrathymic injections of MBP or autogeneic brain cell suspensions can decrease pro-inflammatory cytokine IL-2 concentrations. However, IL-2 concentrations did not significantly decrease upon treatment with MBP or autogeneic brain cell suspensions when administered via the thymus. Compared with Group B2, IL-2 concentrations was markedly decreased postoperatively in Group C2 and in Group D2 at 7 and 14 d (*P* < 0.05). Moreover, Group C2 and Group D2 were significantly different on the 7^th^ and 14^th^ days (*P* < 0.05) (Fig. 1B). This result showed that hepatic portal vein injections of MBP or autogeneic brain cell suspensions decrease pro-inflammatory cytokine IL-2 concentrations. Moreover, autogeneic brain cell suspensions were better than MBP in decreasing IL-2 concentrations when administered via the hepatic portal vein.

#### One-way ANOVA

##### Serum anti-inflammatory cytokine IL-4 concentration

IL-4 concentrations were significantly increased postoperatively in Group C1 and in Group D1 at 3, 7 and 14 d compared with Group B1 (*P* < 0.05). However, IL-4 concentration did not significantly increase in Group C1 or Group D1 (*P* > 0.05) (Fig. 1C). This result indicated that intrathymic injections of MBP or autogeneic brain cell suspensions can promote the concentration of IL-4. However, MBP and autogeneic brain cell suspensions did not significantly promote IL-4 when administered via the thymus. IL-4 concentrations were markedly increased postoperatively in Group C2 at 7, 14 and 21 d and in Group D2 at 3, 7, 14 and 21 d compared with Group B2 (*P* < 0.05). In addition, Group C2 and Group D2 exhibited significant differences postoperatively at 3, 7, 14 and 21 d, (*P* < 0.05) (Fig. 1D). This result showed that hepatic portal vein injections of MBP or autogeneic brain cell suspensions can raise IL-4 concentrations. Moreover, autogeneic brain cell suspensions were better than MBP in raising IL-4 concentrations when injected via the hepatic portal vein.

#### Independent Samples t Test

##### Serum pro-inflammatory cytokine IL-2 concentration

Group C1 had no significant difference from Group C2 (*P* > 0.05) (Fig. 1E). This result revealed that MBP had an equal efficacy on lowering pro-inflammatory cytokine IL-2 concentrations via the thymus and hepatic portal vein. Group D2 significantly differed from Group D1 postoperatively at 3, 7 and 14 d. Furthermore, the pro-inflammatory cytokine IL-2 concentration in Group D2 was lower than in Group D1 (*P* < 0.05) (Fig. 1F). This result demonstrated that hepatic portal vein injections of autogeneic brain cell suspensions had a higher efficacy in decreasing pro-inflammatory cytokine concentrations.

#### Independent Samples t Test

##### Serum anti-inflammatory cytokine IL-4 concentration

Compared with Group C1, Group C2 had a higher concentration of anti-inflammatory cytokine IL-4 at 3, 7 and 21 d after SBI (*P* < 0.05) (Fig. 1G). This result showed that hepatic portal vein injections of MBP were better than intrathymic injections of MBP on increasing the concentration of anti-inflammatory cytokine IL-4. Comparing Group D1 with Group D2 revealed striking significance at 1, 3, 7, 14 and 21 d after SBI. Moreover, the concentration of anti-inflammatory cytokine IL-4 in Group D2 was higher than in Group D1 (*P* < 0.05) (Fig. 1H). This result showed that hepatic portal vein injections of autogeneic brain cell suspensions were better than intrathymic injections of autogeneic brain cell suspensions.

#### Two-way ANOVA

##### Serum pro-inflammatory cytokine IL-2 concentration

In general, when comparing Group II (intrathymic injection group) with Group HPVI (hepatic portal vein injection group), the concentration of IL-2 was significantly different postoperatively at 3, 7 and 14 d. Moreover, the IL-2 concentration of Group HPVI (hepatic portal vein injection group) was lower than Group II (intrathymic injection group) (*P* < 0.05) (Fig. 1I). The results showed that hepatic portal vein injections of brain antigens performed better than intrathymic injections of brain antigens.

#### Two-way ANOVA

##### Serum anti-inflammatory cytokine IL-4 concentration

Significant differences were found between Group II and Group HPVI in the concentration of IL-4 postoperatively at 1, 3, 7, 14 and 21 d. The mean concentration of IL-4 in Group HPVI was higher than in Group II (*P* < 0.05) (Fig. 1J). The results showed that hepatic portal vein injections of brain antigens performed better than intrathymic injections of brain antigens.

### Flow Cytometry

#### One-way ANOVA

**Figure 2:**
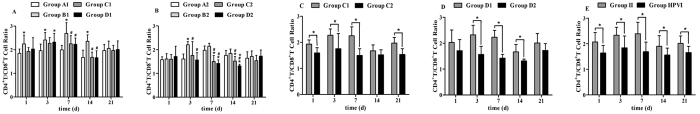
Peripheral blood CD4^+^T/CD8^+^T ratio. In (**A–D**), Group A1: sham, Group A2: sham; Group B1: SBI+ intrathymic injection of normal saline; Group B2: SBI+ hepatic portal vein injection of normal saline; Group C1: SBI+ intrathymic injection of MBP; Group C2: SBI+ hepatic portal vein injection of MBP; Group D1: SBI+ intrathymic injection of autogeneic brain cell suspension; Group D2: SBI+ hepatic portal vein injection of autogeneic brain cell suspension. In (**E**), Group II: intrathymic injection group comprising Group B1 + Group C1 + Group D1; Group HPVI: hepatic portal vein injection group comprising Group B2 + Group C2 + Group D2 . Blood CD4^+^T/CD8^+^T cell ratios that were measured postoperatively at 1, 3, 7, 14 and 21 d. In (**A,B**), *P < 0.05 vs Group A1 (A2); ^#^*P* < 0.05 vs Group B1 (B2); ^+^*P* < 0.05 vs Group C1 (C2). In (**C–E**), *P < 0.05.

The CD4^+^T/CD8^+^T cell ratio in peripheral blood significantly decreased at 7, 14 d after SBI in Group C1 and Group D1 compared with Group B1 (*P* < 0.05). However, no significant difference was found between Group C1 and Group D1 (*P* > 0.05) (Fig. 2A). The results revealed that MBP and autogeneic brain cell suspensions were equally able to decrease the CD4^+^T/CD8^+^T cell ratio via the thymus. Likewise, the cell ratio was considerably decreased at 3, 7 and 14 d after SBI in Group C2 and in Group D2 compared with Group B2. Moreover, the difference between Group C2 and Group D2 was significant at only 14 d (*P* < 0.05) (Fig. 2B). The results revealed that MBP and autogeneic brain cell suspensions were able to decrease the CD4^+^T/CD8^+^T cell ratio, and moreover, autogeneic brain cell suspensions seemed to be better than MBP when administered via the hepatic portal vein.

#### Independent Samples t Test

There were significant differences between Group C1 and Group C2 at 1, 3, 7 and 21 d after SBI. Moreover, the CD4^+^T/CD8^+^T cell ratio in Group C2 were lower than in Group C1 (Fig. 2C). The results showed that hepatic portal vein injections of MBP were able to decrease the CD4^+^T/CD8^+^T cell ratio better than intrathymic injections of MBP. Significant differences were found between Group D1 and Group D2 at 3, 7 and 14 d after SBI (*P* < 0.05). In addition, the CD4^+^T/CD8^+^T cell ratio in Group D2 were lower than Group D1 (*P* < 0.05) (Fig. 2D). The results demonstrated that hepatic portal vein injections of autogeneic brain cell suspensions were better than intrathymic injections of autogeneic brain cell suspension in decreasing the CD4^+^T/CD8^+^T cell ratio.

#### Two-way ANOVA

The differences between Group II and Group HPVI in the CD4^+^T/CD8^+^T cell ratio were significant postoperatively at 1, 3, 7, 14 and 21 d, and the CD4^+^T/CD8^+^T cell ratio of Group HPVI was lower than Group II (*P* < 0.05) (Fig. 2E). The results demonstrated that administration of brain antigens via the hepatic portal vein decreased the CD4^+^T/CD8^+^T cell ratio more efficiently than administration via the thymus.

### Immunohistochemistry

#### One-way ANOVA

**Figure 3:**
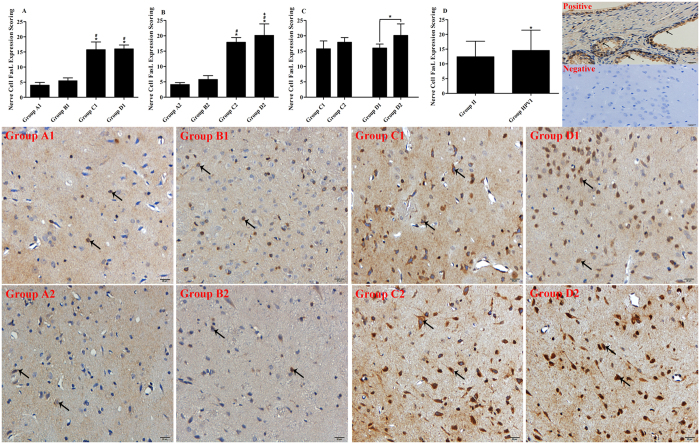
Nerve cell FasL expression. In (**A–C**), Group A1: sham, Group A2: sham; Group B1: SBI+ intrathymic injection of normal saline; Group B2: SBI+ hepatic portal vein injection of normal saline; Group C1: SBI+ intrathymic injection of MBP; Group C2: SBI+ hepatic portal vein injection of MBP; Group D1: SBI+ intrathymic injection of autogeneic brain cell suspension; Group D2: SBI+ hepatic portal vein injection of autogeneic brain cell suspension. In (**D**) Group II: intrathymic injection group comprising Group B1 + Group C1 + Group D1; Group HPVI: hepatic portal vein injection group comprising Group B2 + Group C2 + Group D2. (**A–D**) Show the FasL expression scoring of nerve cells measured at 21 d after SBI. In (**A,B**), *P < 0.05 vs Group A1 (A2), ^#^*P* < 0.05 vs Group B1 (B2), ^+^*P* < 0.05 vs Group C1; In (**C,D**), *P < 0.05. According to the datasheet of the anti-Fas Ligand antibody (Abcam, ab15285), prostate tissue slides were recommended as a positive control. In Fig. 3, positive control shows a prostate tissue slide in which the black arrows indicate positive results, and the negative control shows a slide of brain tissue from a rat. In Fig. 3, immunohistochemistry figures were show nerve cell FasL expression and the scale bars were 20 µm.

The levels of FasL expression in nerve cells in Group C1 and in Group D1 were greater than in Group B1 (*P* < 0.05). However, the FasL expression level in nerve cells was not significantly different between Group C1 and Group D1 (*P* > 0.05) (Fig. 3A). The results demonstrated that MBP and autogeneic brain cell suspensions were equally able to increase the expression of FasL when administered via the thymus. Likewise, the significant differences were found in Group C2 and in Group D2 compared with Group B2, and more FasL was expressed in nerve cells in Group D2 than in Group C2 (*P* < 0.05) (Fig. 3B). The results showed that MBP and autogeneic brain cell suspensions were able to increase the expression of FasL, and moreover, autogeneic brain cell suspensions were better than MBP for increasing FasL expression when administered via the hepatic portal vein.

#### Independent Samples t Test

No significant difference was found between Group C1 and Group C2 (Fig. 3C). This result showed that the increased FasL expression induced by MBP was equal for administration via the thymus and the hepatic portal vein. A significant difference was found between Group D1 and Group D2 (*P* < 0.05) (Fig. 3C). According to the results, autogeneic brain cell suspensions were more effective in increasing the expression of FasL when administered via the hepatic portal vein rather than the thymus.

#### Two-way ANOVA

Group HPVI expressed more FasL than Group II on nerve cells, and furthermore, the FasL expression of the Group HPVI was higher than Group II (*P* < 0.05) (Fig. 3D). This result showed that injection of brain antigens to the hepatic portal vein induced a greater increase the expression of FasL in nerve cells than injection to the thymus.

### TUNEL Assay for Nerve Cells

#### One-way ANOVA

**Figure 4:**
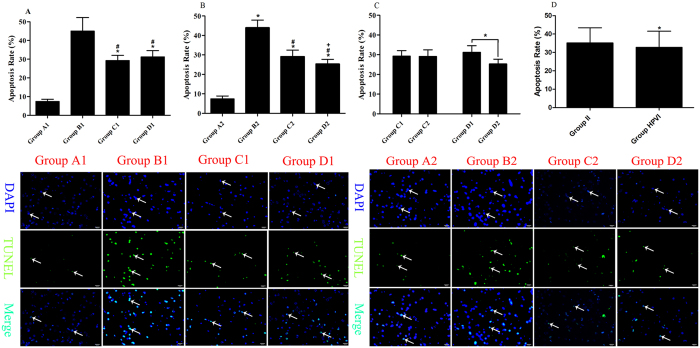
Apoptosis Rate of Brain Sections. In (**A–C**), Group A1: sham, Group A2: sham; Group B1: SBI+ intrathymic injection of normal saline; Group B2: SBI+ hepatic portal vein injection of normal saline; Group C1: SBI+ intrathymic injection of MBP; Group C2: SBI+ hepatic portal vein injection of MBP; Group D1: SBI+ intrathymic injection of autogeneic brain cell suspension; Group D2: SBI+ hepatic portal vein injection of autogeneic brain cell suspension. In (**D**), Group II: intrathymic injection group comprising Group B1 + Group C1 + Group D1; Group HPVI: hepatic portal vein injection group comprising Group B2 + Group C2 + Group D2. (**A–D**) show the apoptosis rates of brain sections measured at 21 d after SBI. In (**A,B**), *P < 0.05 vs Group A1 (A2), ^#^*P* < 0.05 vs Group B1 (B2), ^+^*P* < 0.05 vs Group C1; In (**C,D**), *P < 0.05. In TUNEL figures, DAPI (blue) was used to indicate nuclei (arrows) and TUNEL (green) was used to indicate apoptotic signals (arrows). Merge indicates apoptotic cells (arrows). The scale bars were 20 µm.

The apoptosis rates of nerve cells in Group C1 and Group D1 were lower than in Group B1 (*P* < 0.05). There was no significant difference between Group C1 and Group D1 (*P* > 0.05) (Fig. 4A). These results demonstrated that intrathymic injections of MBP and autogeneic brain cell suspensions equally decreased nerve cell apoptosis rates. The same results were shown in Group C2 and Group D2 as in Group B2, and the apoptosis rate in Group D2 was lower than in Group C2 (*P* < 0.05) (Fig. 4B). These results showed that the apoptosis rates were decreased by hepatic portal vein injections of MBP or autogeneic brain cell suspensions and that autogeneic brain cell suspensions were superior to MBP for decreasing nerve cell apoptosis when administered via the hepatic portal vein.

#### Independent-Samples t Test

There was no significant difference between Group C1 and Group C2 (*P* > 0.05) (Fig. 4C). This result indicated that the capability of MBP in decreasing nerve cell apoptosis was equal when injected via the thymus and the hepatic portal vein. A notable level of statistical significance was found between Group D1 and Group D2 (*P* < 0.05) (Fig. 4C). This result demonstrated that administration of autogeneic brain cell suspensions via the hepatic portal vein was better than via the thymus for decreasing nerve cell apoptosis.

#### Two-way ANOVA

The percentage of nerve cell apoptosis was lower in Group HPVI than in Group II (*P* < 0.05) (Fig. 4D). Injecting autogeneic brain cell suspensions via the hepatic portal vein possessed a higher capability for decreasing the apoptosis of nerve cells than via the thymus.

### Neurological Function Scoring

#### One-way ANOVA

**Figure 5:**
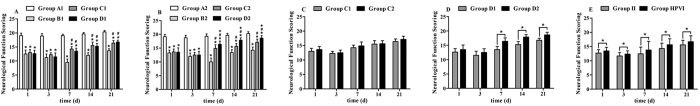
Neurological Function Scoring. In (**A–D**), Group A1: sham, Group A2: sham; Group B1: SBI+ intrathymic injection of normal saline; Group B2: SBI+ hepatic portal vein injection of normal saline; Group C1: SBI+ intrathymic injection of MBP; Group C2: SBI+ hepatic portal vein injection of MBP; Group D1: SBI+ intrathymic injection of autogeneic brain cell suspension; Group D2: SBI+ hepatic portal vein injection of autogeneic brain cell suspension. In (**E**), Group II: intrathymic injection group comprising Group B1+ Group C1  + Group D1; Group HPVI: hepatic portal vein injection group comprising Group B2 + Group C2 + Group D2. Neurological scores were measured postoperatively at 1, 3, 7, 14 and 21 d. In (**A,B**), *P < 0.05 vs Group A1 (A2); ^#^*P* < 0.05 vs Group B1 (B2); ^+^*P* < 0.05 vs Group C1 (C2). In (**C,D**), *P < 0.05.

Compared with Group B1, the neurological scores were notably increased in Group C1 and in Group D1 at 7, 14 and 21 d after SBI (*P* < 0.05). However, there was no significant difference between Group C1 and Group D1 (*P* > 0.05) (Fig. 5A). These results demonstrated that intrathymic injections of MBP or autogeneic brain cell suspensions equally increased neurological scores. The neurological scores were notably increased in Group C2 and Group D2 compared with Group B2 at 7, 14 and 21 d after SBI (*P* < 0.05). Additionally, the neurological scores in Group D2 were higher than in Group C2 at 7, 14 and 21 d after SBI (*P* < 0.05) (Fig. 5B). These results demonstrated that autogeneic brain cell suspensions performed better than MBP in promoting neurological scores when injected via the hepatic portal vein.

#### Independent-Samples t Test

There was no significant difference between Group C1 and Group C2 (*P* > 0.05) (Fig. 5C). The result indicated that the capability of MBP in promoting the recovery of neurological function was equal when injected via the thymus and the hepatic portal vein. Significant differences were found between Group D1 and Group D2 at 7, 14 and 21 d after SBI (*P* < 0.05) (Fig. 5D). These results demonstrated that autogeneic brain cell suspensions injected via the hepatic portal vein were better than injection via the thymus for promoting the recovery of neurological function.

#### Two-way ANOVA

Comparing Group II with Group HPVI, the neurological scores were significantly different postoperatively at 1, 3, 7, 14 and 21 d (*P* < 0.05) (Fig. 5E). The injection of antigens via the hepatic portal vein promoted better recovery of neurological functions than via the thymus.

## Discussion

Cellular inflammatory reactions caused by traumatic brain injury, cerebral infarction and intracranial surgery could worsen cerebral edema, which leads to irreversible neurological deficits. Studies have shown that the control of secondary immune damage can significantly reduce the volume of cerebral edema^7^. Our study demonstrated that injections of MBP or autogeneic brain cell suspensions either via thymus or hepatic portal vein can decrease the levels of pro-inflammatory cytokine IL-2, increase anti-inflammatory cytokine IL-4, degrade the CD4^+^T/CD8^+^T cell ratio and apoptosis of regions of the brain, and up-regulate FasL expression in nerve cells to promote the recovery of neurological functions.

First, we confirmed the establishment of immune tolerance. Autoimmune responses involve various immune cells, especially CD4^+^T helper cells. CD4^+^T cells classically differentiate into two subgroups: pro-inflammatory Th1 cells and anti-inflammatory Th2 cells^19^. Th1 cells mainly secrete IL-2 and IFN-γ, whereas Th2 cells mainly secrete IL-4 and IL-5^20^. Cytokines are necessary regulators for lymphocyte trafficking. It is essential to turn an innate immune response into an adaptive response. IL-2 and other cytokines promote cellular immunity and those cytokines are important for attracting CD4^+^T cells and CD8^+^T cells to the injury site to establish specific immune responses^21^. IL-2 is secreted by activated T cells, especially Th1 cells, and IL-2 itself is an activator of T lymphocytes^22^. Thus, the expression level of IL-2 reflects the degree of activation of Th1 cells. As shown in *in vitro* and *in vivo* studies, IL-2 is capable of breaking immune tolerance^23^. In addition, the central hallmark of anergy is the inability of CD4^+^Th1 clones to synthesize IL-2, which results in abortive proliferative responses and a deficit of producing inflammatory mediators, and thus, IL-2 is an effective and complex balancing factor that affects tolerance and immunity^24^. IL-4 is an essential anti-inflammatory factor and is the characteristic cytokine of Th2 cells that induces the differentiation of Th2 cells^25^. Moreover, Walsh *et al*. indicated that IL-4 mediates neuroprotection and recovery of the injured CNS^26^. T cells serve as a vital part of the immune system, specifically CD4^+^T and CD8^+^T cells. Yilmaz *et al*. found that cerebral ischemia/reperfusion injury significantly increased the level of CD4^+^T cells in cerebral tissue, whereas it decreased the level of CD8^+^T cells. Moreover, the CD4^+^T/CD8^+^T ratio was notably decreased upon drug intervention^27^. Another study also demonstrated that the CD4^+^T/CD8^+^T cell ratio increased in cerebral ischemia/reperfusion injury sites^28^. In experimental stroke, CD4^+^T lymphocytes and CD8^+^T lymphocytes contribute to inflammation, brain injury, and neurological deficit. Consistent with these studies, the expression of IL-2 was decreased, and the expression of IL-4 was increased, which indicated MBP and autogeneic brain cell suspensions led to Th1/Th2 deviation and T cells anergy. The above results, combined with the decreased CD4^+^/CD8^+^T cell ratio, are favorable for the establishment of immune tolerance.

Second, we evaluated the therapeutic effect after the establishment of immune tolerance. Fas ligand (FasL) is capable of inducing apoptosis by combining with Fas specificity. FasL is a type II conserved membrane protein composed of 280 amino acids that belongs to the tumor necrosis factor family. Inactivated T cells express low levels of Fas; otherwise, activated T cells notably up-regulate the expression of Fas. In immunologically privileged organs, such as the eye, brain, and placenta, the inflammatory response is physiologically limited due to the expression of a minute amount of FasL, which induces target cell apoptosis^29^. In the field of transplantation, the expression of FasL in grafts can induce the apoptosis of lymphocytes, thereby protecting the grafts from immune attack^30^,^31^. According to Akiyama K. *et al*.^32^, Fas-expressed T cells can be induced to undergo apoptosis by the systemic infusion of bone marrow mesenchymal stem cells, which express FasL. Our results demonstrated that the levels of FasL expression were higher than in a control group in brain sections, which indicated that the injection of MBP or autogeneic brain cell suspensions into the thymus or hepatic portal vein can up-regulate the expression of FasL in nerve cells. Therefore, up-regulated FasL can protect nerve cells from apoptosis and reduce nerve damage caused by secondary inflammation reactions.

TUNEL assay: In our study, the percentage of apoptosis was lower than in the control group. Namely, the injection of MBP or autogeneic brain cell suspensions into the thymus or hepatic portal vein can induce immune tolerance to protect nerve cells from apoptosis. It was the therapeutic interventions that resulted in protection.

Neurological scores directly reflect the recovery of neurological function. In clinical studies, neurological scoring systems can be used to assess neurological functions. Neurological scoring systems can be used to assess patients who have suffered traumatic brain injury, cerebrovascular disease or intracranial tumors. In our study, a neurological scoring system served as an indispensable tool for assessing the degree of injury and rehabilitation. Our results demonstrated that the scores were higher in the treatment groups; namely, injection of MBP or autogeneic brain cell suspensions into the thymus or the hepatic portal vein promoted the recovery of neurological functions.

These results strongly demonstrated that thymic and hepatic portal vein injections of brain antigens can induce immune tolerance. Intrathymic injections of MBP or autogeneic brain cell suspensions contributed equally in reducing the degree of secondary inflammatory reactions. However, hepatic portal vein injections of autogeneic brain cell suspensions were better than MBP in inducing immune tolerance and decreasing secondary inflammatory reactions. Intrathymic and hepatic portal vein injections of MBP had equal effects on reducing secondary inflammatory reactions. Otherwise, hepatic portal vein injections of autogeneic brain cell suspensions were better than intrathymic injections of autogeneic brain cell suspensions in reducing secondary inflammatory reactions. Moreover, the benefits of injecting antigens into thymus were outweighed by hepatic portal vein injections. Reasons for these differences may be the following: 1. In adult animals, thymuses may undergo atrophy and result in dysfunction. Furthermore, not all brain antigens are exposed in the thymus due to the lack of various digestive enzymes. Therefore, MBP and autogeneic brain cell suspensions had equal efficacies for inducing immune tolerance and lowering secondary inflammatory reactions when administered via the thymus. 2. The liver contains a variety of digestive enzymes that can expose the antigens located in autogeneic brain cell suspensions. Moreover, autogeneic brain cell suspensions are composed of various antigens that can trigger bystander suppression and exert cooperativity. According to a previous study, bystander suppression was activated by tolerogen and resulted in the suppression of immune responses to other antigens^33^. Another study found that experimental arthritis can be prevented by mucosal administration of microbial peptide via bystander suppression^34^. Therefore, hepatic portal vein injections of autogeneic brain cell suspensions were better than a single-antigen MBP for inducing immune tolerance and decreasing secondary inflammatory reactions. 3. To maintain the stability of the internal milieu, the liver must remain tolerant to antigens, such as various products of digested food and metabolic products. Moreover, the liver is characterized by stable immune function and is abounded with immune cells. Therefore, these are the physiological foundations that explain why hepatic portal vein injections surpassed intrathymic injections of MBP or autogeneic brain cell suspensions for decreasing secondary inflammatory reactions.

Third, the mechanisms of immune tolerance must be emphasized. A preliminary study by our group confirmed that immune tolerance can be induced by intrathymic injections of brain antigens^6^. We then confirmed that intrathymic injections of brain antigens can induce immune tolerance by the re-education function of the thymus^35^. In that study, T cells were isolated from the spleens of C57BL/6 mice after intrathymic injection of MBP in the experimental group. The T cells were co-cultured with BV-2 microglia cells in the presence of MBP. Compared with the control group, the CD4^+^T/CD8^+^T ratio was reduced, CD154 was down-regulated, and CD152 was up-regulated on T cell surfaces and pro-inflammatory factors (TNF-α, iNOS, IL-1β) in BV-2 cells were decreased in the experimental group. J. Michael Gee *et al*. showed that MBP-specialized immune tolerance by intranasal administration can depress Th1 cells and enhance Th3 cells and Treg cells^36^. According to Ayer *et al*., Treg cells and the balance of pro-inflammation factors/anti-inflammation factors cause neuroprotection^8^. According to Huang *et al*. reported that combined intrathymic and intravenous injections of mesenchymal stem cells can prolong the survival of rat cardiac allografts, which may be associated with down-regulating miR-155 expression, a shift in the Th1/Th2 balance, and up-regulation of Treg cells expression^9^. Li F *et al*. reviewed that the liver can served as a “school”. In that school, liver APCs serve as “teachers” who “educate” circulating immune cell “students” to inducing immune tolerance^16^. In a study by Lüth S *et al*., the ectopic expression of MBP in mouse livers generated autoantigen-specific CD4^+^CD25^+^Foxp3^+^Tregs^37^. Horst AK *et al*. reviewed that conventional and nonconventional antigen-presenting cells initiate T cells in liver. Immune tolerance can be induced by immune deviation, the induction of T-cell anergy or apoptosis, and the generation and expansion of regulatory T cells in this process^38^. In our study, we found that the CD4^+^T/CD8^+^T ratio was notably decreased and that the Th1/Th2 ratio was deviated.

In recent years, methods of inducing immune tolerance have mainly focused on nasopharynx instillation^8^ or oral administration^39^,^40^. Although these methods are effective in clinical research and animal experiments, they have some disadvantages. For instance, 1. When inducing immune tolerance by oral and nasal mucosal infusion, antigens are absorbed by mucosa, and therefore, some issues cannot be accounted for, such as individual differences among patients and absorptivity, and the amount of antigen cannot be controlled. Furthermore, most patients with severe brain injury suffer from conscious disturbance, and therefore, they have poor compliance. In addition, stress ulcers, a common complication of traumatic brain injury, may prevent the intestinal mucosa from presenting antigens. 2. Patients with brain injury need to fast and maintain an indwell nasal feeding tube that limits the induction of nasal mucosal tolerance, especially in patients who are suffering from infectious coryza or nasal compound injury. Accordingly, we believe that both intrathymic injections and hepatic portal vein injections are vital methods for inducing tolerance. Moreover, this is the first attempt to induce immune tolerance via hepatic portal vein injection of antigens to treat secondary inflammatory reactions caused by SBI. Moreover, we studied the thymus puncture procedure under B ultrasound guidance and found that the injection of antigens into the thymus is safe, efficient and minimally invasive (unpublished). Tuckett AZ *et al*. also reported that B ultrasound is beneficial for use in puncture procedures due to its safety and convenience^41^. Moreover, hepatic portal vein injection of antigens is an efficient way to induce tolerance. Laparoscopic operations have become minimally invasive and mature among general surgery departments. Therefore, treating SBI by inducing immune tolerance to reduce secondary inflammatory reaction by injecting antigens into the thymus or hepatic portal vein are novel methods that pave the way for the treatment of traumatic brain injury, SBI and stroke.

## Methods

### SBI model design and injection of antigens

This protocol was approved by Tianjin Key Laboratory of Cerebral Vascular and Neurodegenerative Diseases, China. Sixty-four male Sprague-Dawley rats (grade SPF; 270–300 g) (Experimental Animal Center of Academy of Military Medical Sciences, China) were randomly divided into 8 groups with 8 rats per group. A standardized SBI model was used according to previous reports^8^,^42^. Briefly, before surgery, general anesthesia was induced via intraperitoneal injection of 0.3 ml/100 g chloral hydrate. The head and manubrium sterni areas of the rats were shaved. Next, each rat was fixed in a stereotaxic frame and the sterile skin on the skull was incised along the biparietal suture through a single sagittal incision. A small square of skull (4 mm diameter) was thinned and removed with a bone drill on the right skull bone 2 mm along the sagittal suture and 1 mm along coronal suture. A durotomy was performed, and 2 mm × 3 mm brain tissue was excised by sharp dissection. Hemostasis was confirmed, and then the skin was closed. Sham groups were operated in the same way but without durotomy. The brain tissues of rats in Group D1 and Group D2 were used for brain cell suspensions, and the brain tissues from rats in the other groups were discarded. Immediately after SBI, the rats were fixed in the supine position, the sterile skin on the surface of the middle of the anterior cervical triangle to the manubrium sterni was incised down to the hypodermis to expose the thymus in Group A1 (sham), Group B1 (SBI+ intrathymic injection of normal saline), Group C1 (SBI+ intrathymic injection of MBP) and Group D1 (SBI+ intrathymic injection of autogeneic brain cell suspension). Then, both sides of the thymus were injected with 50 μl normal saline, MBP or autogeneic brain cell suspensions (except the sham group). Likewise, the hepatic portal vein was exposed in Group A2 (sham), Group B2 (SBI+ hepatic portal vein injection of normal saline), Group C2 (SBI+ hepatic portal vein injection of MBP) and Group D2 (SBI+ hepatic portal vein injection of autogeneic brain cell suspension) and was injected with 100 μl normal saline, MBP or autogeneic brain cell suspension (except the sham group). After injection, hemostasis was confirmed, and then the skin was closed.

### Antigen preparation

#### Brain cell suspension

Under sterile conditions, brain tissue was granulated into single cell suspensions and was thoroughly mixed with 5 ml normal saline. This solution was centrifuged (Beckham) for 5 min at 1000 rpm/min, and the upper liquid was discarded and mixed with 2 ml normal saline. This solution was filtered through 200 mesh filter and divided into two EP tubes in which one tube was cleaved via a chemical lysis method and diluted with normal saline to the concentration equal to the MBP solution, namely, 1 mg/ml. The other tube of solution was diluted with the same multiples (the concentration of brain cell suspension was approximately 2 × 10^5^/ml after dilution).

### Serum Cytokine Concentrations

After induction of general anesthesia, rats were fixed with supine position and blood sampling by cardiac puncture was used to sample 1 ml blood that was injected into promoting coagulation tube. Followed by 30 minutes’ standing at room temperature, the blood sample was centrifuged with 3500 rpm/min, 15 min. The serum was shifted to EP tube and stored at −80 °C. Serum IL-2 and IL-4 concentrations were measured at 1, 3, 7, 14 and 21 d postoperatively according to ELISA kit protocol (Boster Biological Technology, Wuhan, China).

### Flow Cytometry

The protocol of above mentioned blood sampling was as follows. All antibodies were purchased from BioLegend. Antibodies were directly marked with one of the following fluorescent tags: fluorescein isothiocyanate (FITC), phycoerythrin (PE), or allophycocyanin (APC). Anti-CD3, anti-CD4, anti-CD8a antibodies and isotypes were used to react with rat antigens. Cell surface phenotype and sorting were recognized using a flow cytometer (BD biosciences), and the data were analyzed using FlowJo 7.6.1 software.

### Immunohistochemistry

Rats were anesthetized and sacrificed by perfusing through the heart with 4% paraformaldehyde in PBS (pH 7.4). Brains were removed, postfixed overnight and sectioned by the Department of Pathology, Tianjin Huanhu Hospital. The sections were pretreated with xylene and then incubated with primary antibody against rat Fas ligand (Abcam Anti-Fas Ligand antibody, ab15285), at 4 °C overnight. Next, sections were incubated with complement, HRP conjugate, and DAB, orderly (Abcam EXPOSE Mouse and Rabbit Specific HRP/DAB Detection IHC kit, ab80436). Next, sections were counterstained with hematoxylin. Images were captured by a microscopy (Olympus).

The total FasL immunostaining score was computed according to Wang Y *et al*.^43^. Briefly, the percentage of positive was defined as 0 (<5%, negative), 1 (5–25%, sporadic), 2 (25–50%, focal), or 3 (>50%, diffuse). Staining intensity was defined as 0 (no staining), 1 (weak staining), 2 (moderate staining), or 3 (strong staining). The total score of immunostaining was computed as the percentage positive score × staining intensity score, and ranged from 0 to 9. We examined five successive fields as a section and summed the scores.

### TUNEL Assay

Sections were prepared as above. The sections were deparaffinized and rehydrated with xylene and then permeabilized with proteinase K solution. Next, the sections were reacted with TdT reaction buffer, TdT reaction cocktail, orderly. Then they were incubated with Andy Fluor™ 488-Streptavidin staining solution and counterstained with DAPI. Finally, they were covered with antifade mounting medium. Images were captured by a fluorescence microscopy (Olympus). TUNEL kit, DAPI and antifade mounting medium were purchased from GeneCopoeia Inc., Sigma Life Science, and ZSGB-BIO Company, respectively.

The percentage of apoptosis was calculated by the formula: apoptosis rate (%) = (TUNEL-positive nuclei)/(total cell number) × 100%^44^.

### Neurological Function Scoring

An independent researcher who was blinded to experimental designs practiced the modified Garcia scoring system. This scoring system was used to evaluate neurological function in the rats at 1, 3, 7, 14 and 21 d after surgery, as previously described^8^. Briefly, the maximum score of this scoring system is 21 points based on sensorimotor performances ranging from 0 to 3 for the following 7 areas: spontaneous activity, side stroking response, vibrissae response, limb symmetry when the tail was suspended, lateral turning when the tail was suspended, symmetry of walking on the forelimbs when the tail was partially suspended, and climbing ability/response. Neurological function scores were defined as follows: 0 (complete deficit), 1 (definite deficit with some function), 2 (mild deficit or decreased response), or 3 (no evidence of deficit/symmetrical responses). Higher scores correspond to better function and vice versa.

### Statistical Analysis

Date were presented as means ± SD. Statistical analyses were performed with SPSS software (version IBM SPSS Statistics 22.0) and cartograms were drew with GraphPad Prism software (version 5.01). One-way ANOVA and LSD test were used to determine the significance of differences among subgroups. Independent-Samples t Test was used to compare the differences between Group C1 (SBI+ intrathymic injection of MBP) and Group C2 (SBI+ hepatic portal vein injection of MBP), between Group C2 (SBI+ hepatic portal vein injection of MBP) and Group D2 (SBI+ hepatic portal vein injection of autogeneic brain cell suspension). Two-way ANOVA with Bonferroni post hoc test were performed for multiple comparisons (Namely: thymus and hepatic portal vein served as two factors; normal saline, MBP and autogeneic brain cell suspension served as three levels). *P* < 0.05 was considered significant.

## Additional Information

**How to cite this article**: Yang, W. *et al*. Treatment of surgical brain injury by immune tolerance induced by intrathymic and hepatic portal vein injection of brain antigens. *Sci. Rep.* **6**, 32030; doi: 10.1038/srep32030 (2016).
